# Behavioral risk factor clusters among university students at nine universities in Libya

**DOI:** 10.3934/publichealth.2018.3.296

**Published:** 2018-08-10

**Authors:** Walid El Ansari, Khalid A Khalil, Derrick Ssewanyana, Christiane Stock

**Affiliations:** 1 Department of Surgery, Hamad General Hospital, Hamad Medical Corporation, Doha, State of Qatar; 2 College of Medicine, Qatar University, Doha, State of Qatar; 3 School of Health and Education, University of Skövde, Skövde, Sweden; 4 Faculty of Applied Sciences, University of Gloucestershire, Gloucester GL2 9HW, UK^#^; 5 Faculty of Medical Technology, Misrata, Libya; 6 Utrecht Centre for Child and Adolescent Studies, Utrecht University, Netherlands; 7 Unit for Health Promotion Research, Institute of Public Health, University of Southern Denmark, Niels Bohrs Vej 9-10, 6700 Esbjerg, Denmark

**Keywords:** university students, gender, risk factors, health behaviors, cluster analysis

## Abstract

**Objectives:**

This study identifies and describes the clustering of 5 behavioral risk factors (BRFs) among university students. We also investigated whether cluster membership is associated with the students' self-rated academic performance and self-rated health.

**Material and methods:**

A sample of 1300 undergraduates at 6 universities and 3 colleges in Libya completed a self-administered questionnaire that assessed BRFs (nutrition, physical activity, alcohol consumption, smoking, illicit drug use, inadequate sleep). A two-step cluster analysis generated student clusters with similar lifestyles.

**Results:**

Two contrasting clusters of almost even size emerged (after exclusion of alcohol and illicit drug use due to very low prevalence). Cluster 1 comprised students with higher engagement in all forms of physical activity, higher levels of health consciousness, greater daily fruit/vegetable intake and better sleep patterns than students in cluster 2. Only as regards the consumption of sweets, cluster 1 students had less favorable practices than cluster 2 students. The prevalence of smoking was equally low in both clusters. Students in cluster 2, depicting a less healthy lifestyle, were characterized by a higher proportion of women, of students with less income and of higher years of study. Belonging to cluster 2 was associated with lower self-rated health (OR: 0.46, *p* < 0.001) and with lower self-rated academic performance (OR: 0.66, *p* < 0.001).

**Conclusion:**

Preventive programs should not address BRFs in isolation and should particularly target students with clustering of BRFs using specifically tailored approaches.

## 1. Introduction

Modifiable behavioral risk factors (BRFs), such as physical inactivity, tobacco use and unhealthy dietary habits have increased over the years among children and adults across the Arab world [1]. In the context of the present study, BRFs are “detrimental actions that heighten the odds of illness or impede recovery” [2]. Of the 22 Arab countries, Libya had a high prevalence of BRFs, exemplified by the fourth highest prevalence of daily smoking (22.7%) among individuals aged ≥ 15 years [1]. Similarly, Libyan school adolescents exhibited the highest prevalence of overweight (26.6%) in a study of 7 Arab nations [3]. Libyan young people (14–21 years) had high occurrence of sedentary lifestyle, early onset of smoking, and unhealthy dietary habits [4]. Their dietary habits were characterized by elevated sodium intake; over dieting on four meals per day (24.3% of women; 14.6% of men); daily consumption of sweets (> 50% of female adolescents); and high consumption of soft drinks [4]. Equally, Libyan university students had low physical activity (PA) and social engagement, as well as and high occurrence of smoking, over dieting and illicit drug use [5].

Whilst 60% of deaths across the Arab countries result from non-communicable diseases (NCD), few studies have explored the clustering of BRFs among young people of this region [1]. To this end, the university environment is an important setting where patterns and impacts of BRFs among the youth can be explored; and opportunities can be identified for the prevention of such risk factors, as well as for the promotion of health [6]. Hence, understanding BRFs among Libyan university students is opportune. At the time of the present study, ≈20% of the 1.7 million young Libyans were attending university [7], reflecting that universities harbor a considerable proportion of youth in this country.

The Libyan higher education system faces structural and financial constraints [8], and this results in high burdens of psychosocial and health challenges of students (e.g. stress, depression, emotional-cognitive challenges to cope with transition at university) [5],[9]. These characteristics highlight the need for research and interventions targeted at improving student health. The university setting is also an important environment to explore BRFs because students develop independence and life skills largely by experimenting or reinforcing behavioral choices within this setting [6]. Furthermore, BRFs among students are associated with immediate (e.g. decreased self-reported health, lower academic achievement) and longer term (higher risks of morbidity) outcomes [10],[11]. Universities also have various services and organization-wide practices that can potentially benefit the health of students [6], and such services can be optimized if students' behavioral patterns are better understood. In addition, extreme hot weather conditions, political instability, unsafe neighborhoods and sociocultural barriers like social isolation and conservative cultural beliefs in Libya can potentially increase the students' vulnerability to BRFs [1], and those factors have also been linked to decreased PA among young people [12]–[14]. Likewise, aggressive marketing which leads to rise in sales and consumption of unhealthy food commodities, tobacco and alcohol in low- and middle-income countries can increase university students' vulnerability to BRFs [15].

To date, very few studies assessed BRFs among students or adolescents in Libya, and research that undertook such tasks reported the BRFs in isolation from one another [4],[5],[16], despite the tendency for co-occurrence of BRFs [17]–[19]. Clustering of BRFs refers to an observed proportion of a combination of risk factors in excess of its expected proportion [20],[21]. Hence, evaluating the burden of co-occurrence (clustering) of BRFs is important, as such patterns impact adversely on health, social welfare, academic performance and co-morbidities [22]. Understanding the clustering of BRFs therefore has significant implications for the design of prevention strategies and intervention approaches [23]. There seem to be no published studies on BRFs that explored the clustering patterns of health detrimental behaviors among university students in Libya.

This current study bridges the knowledge gap by utilizing a cluster analysis approach [24], an increasingly popular method in the assessment of BRFs. Such analysis involves segmenting a population into mutually exclusive clusters where each cluster contains items with similar patterns or properties, but dissimilar to those in different clusters [25]. Cluster analytic approaches infer from participants' varying responses in the data to classify participants into more homogeneous groups, as opposed to relying on central tendencies and simple interactions which may be inconsistent with the diversity and ecological context of behavior within a community [26]. Thus, cluster analysis is considered as more person-centered and of stronger methodological rationale compared to the traditional variable-centered approaches such as regression models, and despite this, the traditional approaches have been more commonly applied in BRFs research [26],[27].

The main purpose of the current study was to identify and describe the clustering of 5 BRFs (dietary behavior, physical activity, health awareness, sleep, and tobacco smoking) among students at 9 universities/colleges in Libya. We also investigated whether cluster membership was associated with the students' self-rated academic performance and self-rated health.

## 2. Materials and methods

### 2.1. Sampling and data collection

Data were collected as part of the General Student Health Survey conducted in the Eastern Mediterranean Region countries, United Kingdom and other European countries [16],[28],[29]. In Libya, a convenient sample of undergraduate students across 9 institutions (6 universities, 3 colleges) were surveyed in 2008–2009. The inclusion criterion was that a participant was a student at the institution, hence all students at the participating institutions were eligible to partake in the study.

The participating institutions, situated in the 7 cities of Al Bida, Benghazi, Misurata, Sabha, Zawea, Sirt and Tripoli approved the study through their research ethics committees. Students from various courses/academic programs were invited to participate following a detailed explanation of the study (its aims and objectives) and attaining their consent. The participants were mainly from faculties of Engineering, Agriculture, Business, Education, Medical Sciences, and Medical Technology. A self-administered questionnaire was distributed during classroom sessions and students' participation was voluntary and anonymous. Confidentiality was also ensured during the collection of the completed questionnaires. Data were entered, cleaned and stored in a password-secured computer. Out of a total of 2100 students who were provided with the questionnaire, 1567 (74.6%) completed it. We excluded those with missing data in any of the relevant study items, and a total of 1300 fully completed questionnaires were used for the current analysis.

### 2.2. Questionnaire

The data collection tool assessed general health and well-being, social and demographic characteristics, academic achievement, and behavioral characteristics. The questionnaire items were based on previous student health surveys [16],[28],[29] and were translated from English to Libyan language through two independent translations, which were compared for any inconsistencies. In addition to the socio-demographic characteristics (university/college attended, year of study, age, sex, religion and type of accommodation during academic term), the following BRF items were used in the present study:

*Self-rated health*: “How would you rate your health in general?” (5-point response options: 1 = poor, 2 = fair, 3 = good, 4 = very good, 5 = excellent). The item was taken from the US American College Health Association National College Health Assessment [30].

*Self-rated academic performance*: “How do you rate your performance in comparison with your fellow students?” (5-point response options: 1 = much worse, 2 = worse, 3 = the same, 4 = better, 5 = much better). The item was taken from the UK Student Health Survey [28].

*Health awareness/consciousness*: “To what extent do you keep an eye on your health?” (Response on 4 point scale: 1 = not at all, 4 = very much). The item was taken from the German Youth Health Survey [34] in its English version as previously used by El Ansari and Stock et al. in university students in the UK Student Health Survey [28].

*Vigorous physical activity*: “During the last 7 days, on how many days did you do vigorous physical activities like lifting, digging, aerobics or fast cycling that last at least 10 minutes and makes you breathe harder than normal?” (Respondents specified the number of days, 0–7). This was according to the measure and item formulation developed by the American Heart Association [32].

*Moderate physical activity*: “During the last 7 days, on how many days did you do moderate physical activity like carrying light loads, cycling at a regular pace or double tennis for at least 10 minutes? Do not include walking (Moderate activities refers to activities that make you breathe somewhat harder than normal)” (Respondents specified the number of days, 0–7). This was according to the measure and item formulation developed by the American Heart Association [32].

*Muscle strengthening physical activity*: “On how many of the past 7 days did you do exercises to strengthen or tone your muscles, such as push-ups, sit-ups or weight lifting?” (Respondents specified the number of days, 0–7). This was according to the measure and item formulation developed by the American Heart Association [32].

*Fruit/vegetable daily consumption:* “How many servings of fruits and vegetables do you usually have per day (1 serving = 1 medium piece of fruit, ½ cup chopped, cooked or canned fruits/vegetables, ¾ cup fruit/vegetable juice, small bowl of salad greens, or ½ cup dried)?” (4 response options: “I don't eat fruits and vegetables”, “1–2”, “3–4”, “5 or more” servings). The item was taken from the food frequency questionnaire of the German Youth Health Survey [34] in its English version as used by others in research among university students [33].

*Consumption of sweets*: “How often do you eat sweets (chocolate, candy, etc.)?” (5 response options: “Several times a day”, “daily”, “several times a week”, “1–4 times a month”, “never”). The item was taken from the food frequency questionnaire of the German Youth Health Survey [34] in its English version as used among university students [33].

*Importance of eating healthy*: “How important is it for you to eat healthy?” (4 response options: 1 = not at all important, 4 = very important). The item was taken from the food frequency questionnaire of the German Youth Health Survey [31] in its English version as used by among university students [33].

*Smoking (past 3 months)*: “Within the last three months, how often did you smoke? (Cigarettes, pipes, cigarillos, cigars)” (3 response options: “Daily”, “occasionally”, “never”). The item was taken from the food frequency questionnaire of the German Youth Health Survey [34] in its English version as used by El Ansari et al. in university students [35].

*Illicit drugs (ever use)*: “Have you ever use/used drugs?” (3 response options: “Yes, regularly”, “yes, but only a few times”, “never”). The item was taken from the food frequency questionnaire of the German Youth Health Survey [34] in its English version as used by El Ansari et al. in university students [35].

*Alcohol consumption*: “Do you drink alcohol?” (3 response options: “Daily”, “occasionally”, “never”). The item was taken from the food frequency questionnaire of the German Youth Health Survey [34] in its English version as used by El Ansari et al. in university students [35].

*Enough sleep*: “On how many of the past 7 days did you get enough sleep so that you felt rested when you woke up in the morning?” (Respondents specified the number of days, 0–7). The item was taken from the UK Student Health Survey as used previously by El Ansari et al. [35].

*Disposable income*: “Would you say the amount of money you have is…” (3 response options: “Always sufficient”, “mostly sufficient”, “mostly insufficient”, and “always insufficient”). The item was taken from the Cross National Student Health Survey as used previously by Stock et al. [36].

### 2.3. Statistical analysis

Using the statistical package STATA 14, bivariate analyses were conducted to describe the distribution and characteristics of various BRFs among students. This was followed by a two-step cluster analysis [25] conducted in the statistical package SPSS v.23.0 and based on 5 BRFs (dietary behavior, physical activity, health awareness, sleep, tobacco smoking). The variables “alcohol consumption” and “ever use of illicit drugs” were not included as the proportion of abstainers was almost equivalent to 100% among female participants. Two-step cluster analysis was used to identify groupings which differ on criterion variables within a data set and the procedure combines pre-clustering and hierarchical methods [25]. Log-likelihood and Euclidian distance measures were applied in the two-step cluster analysis because the BRFs comprised continuous and categorical variables. Cluster number selection was automated using the Schwarz's Bayesian Criterion (BIC). Within each cluster, for categorical BRFs, the frequency of the categories was presented as percentages; while for continuous BRFs, a mean value (with standard deviation) was presented. Differences in distribution of socio-demographic characteristics and BRFs across clusters were tested by Chi-square tests (for categorical variables) [37] or MANOVA (for continuous variables) [37]. Finally, we conducted two separate ordinal logistic regression models in STATA 14 to investigate the association between the cluster membership and the two outcomes of *self-rated health* and *self-rated academic performance*, while adjusting for participants' sex and institution (the latter in order to adjust for the nested data structure). In all analyses, statistical significance was set at *p* < 0.05.

## 3. Results

### 3.1. Sample characteristics

The sample comprised 1300 students at 6 universities and 3 colleges: Misurata University (previously 7th October University, n = 463, 35.6%); University of Tripoli (previously Al-Fateh University, n = 190, 14.6%); Omar El-Muktar University (n = 88, 6.8%); Sirt University (previously Tahadi University, n = 191, 14.7%); Sebha University (n = 79, 6.1%); Benghazi University (previously Garyounis University, n = 58, 4.5%); Al Mahed Al Senai College (Misurata, n = 72, 5.5%); Mahad Al Zawea College (Al Zawea, n = 88, 6.8%); and Higher Medical Technology Institute (n = 71, 5.5%). The majority of the respondents were women (66.2%) and Muslims (99.9%). Male participants were on average older (21.1, SD = 2.4 years) than their female counterparts (20.9, SD = 2.3 years). Most participants (81.7%) lived with their parents during university term, 13.7% lived with a roommate, and 4.6% stayed with a spouse or lived alone. The majority of students (72.9%) rated their disposable income during university term as mostly or always sufficient, while the rest had insufficient income during term time.

### 3.2. Behavioral risk factors

Table 1 summarizes the gender aggregated characteristics of the BRFs. Fruit and vegetable consumption was equally low among both genders, and only 10.2% achieved the recommended consumption of ≥ 5 fruit/vegetable servings daily [38]. Similarly, daily consumption of sweets (including several times per day) was high (35.2%), and was more common among women. Within the past 3 months, 99.3% of women did not smoke, whereas a quarter of the males either smoked occasionally or on daily basis. Most (98.4%) students had never used illicit drugs (with the exception of 4.6% of men), and only 3.4% (mainly men) had ever drunk alcohol. For all types of PA, men exercised significantly more than women; however with exception of the 2.9 days per week of muscle strengthening among men, none of the levels of the other types of PA achieved by both sexes satisfied the international recommended guidelines [39]. Students reported an average of 3.1 days per week where they felt well rested after sleep (no gender difference). In terms of health consciousness/awareness (to what extent they keep an eye on their health), overall, 80.2% of the sample was highly health conscious (i.e. ranged from “very much” to “to some extent”), and health consciousness was slightly higher among men than women. Similarly, 82.1% of the sample regarded eating healthy as very important or important.

**Table 1.** Gender aggregated characteristics of behavioral risk factors.

Behavioral risk factor	Whole Sample N = 1300 N (%)	Men n = 439 N (%)	Women n = 861 N (%)	*p*-value
*Fruit/vegetable servings (Daily)*				0.453
≥ 5	132 (10.2)	42 (9.6)	90 (10.5)	
3–4 servings	332 (25.5)	113 (25.7)	219 (25.4)	
1–2 servings	757 (58.2)	251 (57.2)	506 (58.8)	
I don't eat fruits/vegetables	79 (6.1)	33 (7.5)	46 (5.3)	
*Consumption of sweets*				< 0.001
Never	56 (4.3)	39 (8.9)	17 (2.0)	
1–4 times a month	239 (18.4)	97 (22.1)	142 (16.5)	
Several times a week	548 (43.1)	188 (42.8)	360 (41.8)	
Daily	304 (23.4)	80 (18.2)	224 (26.0)	
Several times a day	153 (11.8)	35 (8.0)	118 (13.7)	
*Smoking (past 3 months)*				< 0.001
Never	1188 (91.4)	333 (75.9)	855 (99.3)	
Occasionally	49 (3.8)	43 (9.8)	6 (0.7)	
Daily	63 (4.8)	63 (14.3)	0 (0.0)	
*Illicit drug/s (ever use)*				< 0.001
Never	1279 (98.4)	419 (95.4)	860 (99.9)	
Only few times	13 (1.0)	13 (3.0)	0 (0.0)	
Regularly	8 (0.6)	7 (1.6)	1 (0.1)	
*Alcohol consumption*				< 0.001
Never	1256 (96.6)	404 (92.0)	852 (98.9)	
Occasionally	40 (3.1)	31 (7.1)	9 (1.1)	
Daily	4 (0.3)	4 (0.9)	0 (0.0)	
Physical activity* (days/week)				
Vigorous PA for ≥ 10 minutes	1.2 ± 2.0	2.0 ± 2.4	0.8 ± 1.7	< 0.001
Muscle strengthening PA	1.9 ± 2.3	2.9 ± 2.6	1.3 ± 1.9	< 0.001
Moderate PA for ≥ 10 minutes	1.3 ± 2.0	2.1 ± 2.4	0.8 ± 1.7	< 0.001
Enough sleep* (days/week)	3.1 ± 2.3	3.0 ± 2.4	3.2 ± 2.3	0.835
*Health consciousness*				0.005
Very much	418 (32.1)	159 (36.2)	259 (30.1)	
To some extent	625 (48.1)	205 (46.7)	420 (48.8)	
Not much	227 (17.5)	60 (13.7)	167 (19.4)	
Not at all	30 (2.3)	15 (3.4)	15 (1.7)	
*Importance of eating healthy*				0.050
Very important	861 (66.2)	285 (64.9)	576 (66.9)	
2^nd^ to very important	207 (15.9)	68 (15.5)	139 (16.1)	
3^rd^ to very important	124 (9.5)	37 (8.4)	87 (10.1)	
4^th^ to very important	37 (2.9)	20 (4.6)	17 (2.0)	
Not at all important	71 (5.5)	29 (6.6)	42 (4.9)	

* Cell values for these BRFs represent means ± standard deviation.

### 3.3. Clustering of BRFs among students

The analysis generated two clusters of almost even size (ratio of biggest to smallest = 1.09) (Table 2). Except for smoking, there were statistically significant differences in all the BRFs across the two clusters. Cluster 1 comprised students with higher engagement in all PA types, high levels of health consciousness (keeping an eye on one's health), greater daily fruit/vegetable intake and better sleep compared to cluster 2 students. Only their sweets consumption was higher than that of students in cluster 2. Cluster 2 included students who were less physically active and had poorer sleep patterns. Their daily fruit/vegetable consumption was lower and their sweets consumption was overall high, but lower than that of cluster 1 students. Cluster 2 students placed less importance on eating healthy and were less health conscious compared to cluster 2. There was no significant difference between clusters regarding smoking, which had a low prevalence in both clusters.

Statistically significant differences between the clusters in terms of sociodemographic characteristics were evident only for sex, year of study and sufficiency of disposable income (Table 3). Compared to cluster 1, cluster 2 was composed of more women (*p* < 0.001) and more students in higher years of study (i.e. third or fourth year and above, *p* = 0.017). More cluster 1 (75.6%) compared to cluster 2 (70.0%) students rated their disposable income as always or mostly sufficient (*p* < 0.001).

**Table 2.** Behavioral risk factors of two clusters of university students in Libya.

Behavioral risk factor	Cluster 1 (n = 677)	Cluster 2 (n = 623)	*p*-value
*Daily fruit/vegetable servings*			*p* < 0.001, Cramer's phi = 0.37
≥ 5	98 (14.5)	34 (5.5)	
3–4 servings	249 (36.8)	83 (13.3)	
1–2 servings	322 (47.5)	435 (69.8)	
I don't eat fruits and vegetables	8 (1.2)	71 (11.4)	
*Consumption of sweets*			*p* = 0.003, Cramer's phi = 0.11
Never	23 (3.4)	33 (5.3)	
1–4 times/month	115 (17.0)	124 (19.9)	
Several times/week	272 (40.2)	276 (44.3)	
Daily	187 (27.6)	117 (18.8)	
Several times/day	80 (11.8)	73 (11.7)	
*Smoking in past 3 months*			*p* = 0.45, Cramer's phi = 0.03
Never	625 (92.3)	563 (90.4)	
Occasionally	23 (3.4)	26 (4.2)	
Daily	29 (4.3)	34 (5.4)	
Physical activity* (days per week)			
Vigorous PA for ≥ 10 minutes	1.9 ± 2.4	0.5 ± 1.2	*p* < 0.001, λ = 0.97
Muscle strengthening PA	2.8 ± 2.5	0.9 ± 1.6	*p* < 0.001, λ = 0.94
Moderate PA for ≥ 10 minutes	1.8 ± 2.4	0.6 ± 1.4	*p* < 0.001, λ = 0.99
Enough sleep* (days per week)	*3.5 ± 2.4*	*2.7 ± 2.2*	*p* < 0.001, λ = 0.97
*Health consciousness*			*p* < 0.001, Cramer's phi = 0.59
Very much	380 (56.1)	38 (6.1)	
To some extent	268 (39.6)	357 (57.3)	
Not much	26 (3.8)	201 (32.3)	
Not at all	3 (0.4)	27 (4.3)	
*Importance of eating healthy*			*p* < 0.001, Cramer's phi = 0.58
Very important	625 (92.4)	236 (37.9)	
2^nd^ to very important	34 (5.0)	173 (27.8)	
3^rd^ to very important	2 (0.3)	122 (19.6)	
4^th^ to very important	11 (1.6)	26 (4.1)	
Not at all important	5 (0.7)	66 (10.6)	

* Cell values for these BRFs represent means ± standard deviation; λ: Wilks' Lambda statistic from MANOVA.

**Table 3.** Socio-demographic characteristics of the clusters.

Variable	Cluster 1 (N = 677)	Cluster 2 (N = 623)	*p*
*Sex*			< 0.001
Women	417 (61.6)	444 (71.3)	
Men	260 (38.4)	179 (28.7)	
*Year of study*			0.017
First year	250 (36.9)	181 (29.1)	
Second year	175 (25.9)	181 (29.1)	
Third year	151 (22.3)	168 (26.9)	
Fourth year and above	101 (14.9)	93 (14.9)	
*Disposable income*			
Always sufficient	203 (30.0)	119 (19.1)	< 0.001
Mostly sufficient	309 (45.6)	317 (50.9)	
Mostly insufficient	87 (12.9)	121 (19.4)	
Always insufficient	78 (11.5)	66 (10.6)	

### 3.4. Association between behavioral risk factor cluster and self-rated academic performance

When asked to rate their academic performance in comparison to their fellow students, almost half (49.4%) of the sample indicated that their performance was the same, 38% indicated better performance, 6.7% reported much better performance, and 12.4% reported worse performance than their colleagues. Ordinal logistic regression (adjusting for sex) examined the relationship between BRF cluster and academic performance (Table 4). Belonging to cluster 2 was associated with lower odds of being in a higher self-rated academic performance category adjusted for participants' sex (OR: 0.66, *p* < 0.001). In addition, sex was strongly associated with self-rated academic performance, where men had lower odds of high self-rated academic performance (OR: 0.57, *p* < 0.001).

**Table 4.** Association between BRF cluster and self-rated academic performance and health.

	Self-rated			
	Academic performance (n = 1300)		Health (n = 1300)	
*Cluster*	*Odds ratio (95% CI)*	*p*	*Odds ratio (95% CI)*	*p*
Cluster 1	Reference		Reference	
Cluster 2	0.66 (0.54, 0.82)	< 0.001	0.46 (0.38, 0.57)	< 0.001
*Sex*				
Women	Reference		Reference	
Men	0.57 (0.45, 0.72)	< 0.001	0.90 (0.72, 1.24)	0.358

Ordinal logistic regression (adjusted for gender and institution); CI: Confidence interval; Self-rated academic performance: Ordinal outcome with increasing levels (much worse, worse, the same, better, much better); Self-rated health: Ordinal outcome with increasing levels (poor, fair, good, very good, excellent).

### 3.5. Association between behavioral risk factor cluster and self-rated health

The majority of participants (66.2%) self-rated their health between good and very good, while 19.5% rated it as excellent, and 11.8% and 2.5% rated it as fair and poor respectively. As depicted in Table 4, ordinal logistic regression showed that being a student of cluster 2 compared to cluster 1 was associated with lower odds of perceiving one's self-rated health as good (OR: 0.46, *p* < 0.001). Sex was not associated with self-rated health.

## 4. Discussion

The health behaviors of individuals (whether they smoke, how much they drink, what they eat, whether they are regularly physically active) influence their health and mortality risk [40]. A substantial burden of illness is associated with these health behaviors. Less is known about how these behaviors cluster together [41]. We assessed the clustering and co-distribution of lifestyle risk factors, examining 5 major BRFs among Libyan students.

Our analysis yielded 2 BRFs clusters of almost even size; cluster 1 with more favorable lifestyle habits and cluster 2 with mostly less favorable lifestyle patterns. Such 2 cluster findings are consistent with research among Chinese college students (aged ≈ 19.7 years, similar to our mean age of 21.0 years) where a two-step cluster analysis identified 2 different clusters [42]. Likewise, among male adolescents in Saudi Arabia, 6 health-compromising behaviors (including low fruit and high sweet consumptions, low PA, smoking) clustered into 2 conceptually distinct clusters [43]. However, our findings contrast with others who found ≥ 3 distinct clusters of multiple health behaviors among college students [44]. While it is not straightforward to speculate the reasons for such differences, it could be because other studies included additional variables e.g. alcohol consumption (in the USA) [44], illicit drug use (in the UK) [45], or out-of-home eating (in China) [46]. In contrast, our cluster analysis did not include “alcohol consumption” and “ever use of illicit drugs” because abstainers comprised ≈100% among the female participants. This suggests that students from Arab countries potentially exhibit a lower number of clusters, where the low consumption of alcohol and illicit drug use reduces the variation of BRFs among this population.

As for the clusters' socio-demographic characteristics, our healthier cluster 1 comprised more men, compared to the less healthy cluster 2 (*p* < 0.001). This is in contrast to research from Spain on the associations between smoking, high-risk alcohol consumption, leisure-time sedentariness and unbalanced diet, which reported that behavior-related risk factors tended to aggregate, and that such aggregation was higher among men [47]. Our findings also contrast with those from Greece, where although health-compromising behaviors (14 behavioral and metabolic health risk factors) were highly prevalent across both genders, men engaged in more health risk behaviors than women [48]. Likewise, in terms of age, our cluster 2 (compared to cluster 1) had more students in higher years of study i.e. ≥ third or fourth year, suggesting a higher risk among the slightly older students. The contrasts of our findings with those from Spain and Greece [47],[48] might be because: (1) Alcohol use was not included in our analysis; (2) physical inactivity was higher for Libyan women than men; and (3) nutritional behavior and health consciousness were—different from what is commonly seen among European students—not better for women than men. Such gender and age differences in clustering patterns calls for gender specific attention when designing intervention programs, with perhaps more “intensive” strategies required for women in higher years of study.

As for the clusters' components, members of our less healthy cluster 2 were minimally physically active, had poorer sleep patterns, lower daily fruit/vegetable consumption, high sweet consumption, placed lower importance on eating healthy and were less health conscious. Our findings are consistent with those from Switzerland, where multiple risk factors coexisted in a substantial percentage of students [49] and with those among Nigerian adolescents, where the prevalence of clustering of modifiable risk factors (alcohol, physical inactivity, cigarette smoking, poor dietary patterns) was high [50].

Our cluster 2 constellation of BRFs is similar to findings from Ireland, where health behavior cluster analysis revealed groups of physically inactive students (17.8%) and those with multiple risk factors (17%) [10]; and with findings in China, where two-step cluster analysis found an unhealthy (25.7%) and an moderately healthy (31.1%) group [46]. Our cluster 2 findings support the assumption that individuals at a higher level for one BRF were more likely to be also at a higher level for another BRF [51]. The reasons/mechanisms through which health behaviors cluster in some individuals rather than others are not easy to disentangle and may include: “Transfer” effects (e.g. nonsmokers consume less alcohol and regularly active people smoke less) and “compensation” effects (e.g. people who consume alcohol more frequently are more physically active in order to compensate the potential harm related to drinking) [52]. Alternatively, a framework of more generic protective and risk factors might be helpful in exploring students' multiple BRFs. Risk factors that stimulate several risk behaviors simultaneously could be social pressures to use alcohol and tobacco, to consume unhealthy food and to neglect sleep. More generic protective factors, could be values and expectations for academic achievement or support and control from parents, friends, or partners [53]. Other explanations why BRFs cluster in individuals include personality or psychological factors, whereby conscientiousness or extraversion are concomitants of health behaviors among university populations [54]. Future research could explore such propositions.

As for the association between BRFs clusters and self-rated health, we observed that while adjusting for sex, membership to cluster 2 (compared to cluster 1) was associated with lower odds of perceiving one's own health as good (OR: 0.47, *p* < 0.001). Our findings concur with research in Spain, where a clustering of risk factors was associated with a higher frequency of suboptimal perceived health [47]. Thus, compared to persons with none of 4 risk factors (smoking, high-risk alcohol consumption, leisure-time sedentariness, unbalanced diet), individuals who simultaneously reported 3 or 4 risk factors more frequently reported suboptimal subjective health [47]. However, the Spanish study [47] comprised a general population sample with wider age band (18–64 years), while this current study was confined to young adult university students. Certainly, effects of unhealthy BRFs clustering extend beyond self-rated health to actual health, where among Dutch high school adolescents, unhealthy behavior was associated with poor psychosocial and physical health [55]; and in China among those aged ≥ 18 years with hypertension, there was a clear gradient between the number of BRFs and actual blood pressure level for men and women (*p* < 0.05 for both genders) [56].

In terms of the link between BRFs cluster and self-rated academic performance, belonging to cluster 2 (compared to cluster 1) was associated with lower odds of being in a higher self-rated academic performance category even when adjusted for sex (OR: 0.63, *p* < 0.001). Such findings suggest that the BRFs' clustering effects reach far beyond self-rated health and actual disease processes, to influence individuals' academic achievement, a great concern for university populations. Whilst we employed self-reported academic performance, future research could examine this relationship using objective measure/s e.g. module grades achieved by students.

Some limitations need to be considered when interpreting our findings. The cross-sectional study design is not conclusive on causal relationships. As general health surveys need to observe certain limits of respondent burden, some variables were measured by single items. Self-reported data were used and we did not have access to more objective measures of academic achievement, such as the actual grades attained by students. In addition, recall bias, social desirability and sociability may have affected the responses. Our sampling method during lessons/lectures resulted in a high response rate and possibly low self-selection bias, but those not present in class at the time of data collection (potentially due to clustering of BRFs), did not have an opportunity to participate in the survey, and while we tried that the sample is as representative as possible, it remains a convenience sample. The data were collected 9 years ago and the prevalence of BRFs might have undergone changes during this period, which could have resulted in changes in the composition and size of clusters. Although we cannot rule out that changes in student behaviours could have taken place, we do not assume such changes, should they exist, to be of large magnitude and impact regards the resulting two clusters and the associations found with self-rated health and academic achievement. Data from Libya is very scarce, and there does not exist anymore recent comparative data on BRFs among young people from Libya, except for a study published in 2015 on smoking in 25 Eastern Mediterranean and Eastern European countries, but this study also reports Libyan data from 2010 [57].

Despite these limitations, the current study collected data from a large number of universities and colleges in Libya resulting in a high representability. The study provides new insights on a wider range of key BRFs across a large sample of undergraduates across Libya, an under-researched and difficult-to-reach group.

## 5. Conclusion

Our findings suggest the potential for interventions targeting BRFs, either sequentially or concurrently, particularly where there is clustering. A practical implication of this research is the possibility to tailor prevention programs more specifically towards the risk factor profiles of individuals, which could be easily obtained prior to counseling or other educational activities. Any material produced could take into consideration that, among this student population, two quite different groups of students exist, a point that needs to be addressed with respect to tailored preventive strategies, language and programs. In addition, a broader approach to risk behavior prevention is warranted. Behavioral interventions focused on modifying BRFs, that simultaneously address multiple domains of risk and protective factors of risk behavior need to be tested. Such strategies, tailored to students' age and gender, could potentially have larger effects on prevention than those targeting any single behavior in isolation. Likewsie, as behavior does not appear in vacuum, attention to the wider influences on risk behavior at the level of the university environment, as well as at the broader societal level is necessary i.e. culture, media and social climate should as well be considered through broader social policy strategies.

## References

[1] Rahim HFA, Sibai A, Khader Y (2014). Non-communicable diseases in the Arab world. Lancet.

[2] Spring B, Moller AC, Coons MJ (2012). Multiple health behaviours: Overview and implications. J Public Health.

[3] Musaiger A, Al-Mannai M, Tayyem R (2012). Prevalence of overweight and obesity among adolescents in seven Arab countries: A cross-cultural study. J Obes.

[4] Sachithananthan V, Buzgeia M, Awad F (2013). Nutritional status, dietary profile and selected lifestyle attributes of adolescents and early adults in Benghazi, Libya. J Food Nutr Disord.

[5] Salam A, Alshekteria A, Mohammed H (2012). Physical, mental, emotional and social health status of adolescents and youths in Benghazi, Libya. East Mediterr Health J.

[6] Tsouros A, Dowding G, Thompson J (1998). Health promoting universities: Concept, experience and framework for action.

[7] Education Audiovisual and Culture Executive Agency (EACEA) Higher Education in Libya, 2012. http://eacea.ec.europa.eu/tempus/participating_countries/overview/libya_overview_of_hes_final.pdf.

[8] Tamtam A, Gallagher F, Olabi AG (2011). Higher education in Libya, system under stress. Proc Soc Behav Sci.

[9] Ansari WE, Khalil K, Stock C (2014). Symptoms and health complaints and their association with perceived stressors among students at nine Libyan universities. Int J Environ Res Public Health.

[10] Conry MC, Morgan K, Curry P (2011). The clustering of health behaviours in Ireland and their relationship with mental health, self-rated health and quality of life. BMC Public Health.

[11] So ES, Park BM (2016). Health behaviors and academic performance among Korean adolescents. Asian Nurs Res.

[12] Tucker P, Gilliland J (2007). The effect of season and weather on physical activity: A systematic review. Public Health.

[13] Molnar BE, Gortmaker SL, Bull FC (2004). Unsafe to play? Neighborhood disorder and lack of safety predict reduced physical activity among urban children and adolescents. Am J Health Promot.

[14] Abbasi IN (2014). Socio-cultural barriers to attaining recommended levels of physical activity among females: A review of literature. Quest.

[15] Moodie R, Stuckler D, Monteiro C (2013). Profits and pandemics: Prevention of harmful effects of tobacco, alcohol, and ultra-processed food and drink industries. Lancet.

[16] El Ansari W, Khalil K, Crone D (2014). Physical activity and gender differences: Correlates of compliance with recommended levels of five forms of physical activity among students at nine universities in Libya. Cent Eur J Public Health.

[17] Baskin-Sommers A, Sommers I (2006). The co-occurrence of substance use and high-risk behaviors. J Adolesc Health.

[18] Willoughby T, Chalmers H, Busseri MA (2004). Where is the syndrome? Examining co-occurrence among multiple problem behaviors in adolescence. J Consult Clin Psychol.

[19] Zweig JM, Lindberg LD, Mcginley KA (2001). Adolescent health risk profiles: The co-occurrence of health risks among females and males. J Youth Adolesc.

[20] Schuit AJ, van Loon AJM, Tijhuis M (2002). Clustering of lifestyle risk factors in a general adult population. Prev Med.

[21] Costa E, Dias CM, Oliveira L (2014). Clustering of behavioural risk factors in the Portuguese population: Data from National Health Interview Survey. J Behav Health.

[22] Huang DY, Lanza HI, Murphy DA (2012). Parallel development of risk behaviors in adolescence: Potential pathways to co-occurrence. Int J Behav Dev.

[23] Hale D, Viner R (2012). Policy responses to multiple risk behaviours in adolescents. J Public Health.

[24] Scott A, Knott M (1974). A cluster analysis method for grouping means in the analysis of variance. Biometrics.

[25] Kaufman L, Rousseeuw PJ (2009). Finding groups in data: An introduction to cluster analysis.

[26] Rapkin BD, Luke DA (1993). Cluster analysis in community research: Epistemology and practice. Am J Community Psychol.

[27] Lanza ST, Cooper BR (2016). Latent class analysis for developmental research. Child Dev Perspect.

[28] El Ansari W, Stock C (2010). Is the health and wellbeing of university students associated with their academic performance? Cross sectional findings from the United Kingdom. Int J Environ Res Public Health.

[29] El Ansari W, Stock C, Mills C (2013). Is alcohol consumption associated with poor academic achievement in university students?. Int J Prev Med.

[30] American College Health Association (2007). American College Health Association National College Health Assessment (ACHA-NCHA): Spring 2006 Reference Group Report (abridged). J Am Coll Health.

[31] Stock C, Kücük N, Miseviciene I (2002). Differences in health complaints between university students from three European countries. Eur J Public Health.

[32] Haskell WL, Lee IM, Pate RR (2007). Physical activity and public health: Updated recommendation for adults from the American College of Sports Medicine and the American Heart Association. Circulation.

[33] Mikolajczyk RT, El Ansari W, Maxwell AE (2009). Food consumption frequency and perceived stress and depressive symptoms among students in three European countries. Nutr J.

[34] Hurrelmann K, Kolip P (1994). The Youth Health Survey. Public Relations Service, SFB 227, No 11.

[35] El Ansari W, Stock C, John J (2011). Health promoting behaviours and lifestyle characteristics of students at seven universities in the UK. Cent Eur J Public Health.

[36] Stock C, Mikolajczyk R, Bloomfield K (2009). Alcohol consumption and attitudes towards banning alcohol sales on campus among European university students. Public Health.

[37] Mchugh ML (2013). The chi-square test of independence. Biochem Med.

[38] He FJ, Nowson CA, Macgregor GA (2006). Fruit and vegetable consumption and stroke: Meta-analysis of cohort studies. Lancet.

[39] World Health Organization (2010). Global recommendations on physical activity for health. http://www.who.int/dietphysicalactivity/factsheet_recommendations/en/.

[40] Murray CJ, Vos T, Lozano R (2012). Disability-adjusted life years (DALYs) for 291 diseases and injuries in 21 regions, 1990–2010: A systematic analysis for the Global Burden of Disease Study 2010. Lancet.

[41] Buck D, Frosini F (2012). Clustering of unhealthy behaviours over time: Implications for policy and practice.

[42] Ye YL, Wang PG, Qu GC (2016). Associations between multiple health risk behaviors and mental health among Chinese college students. Psychol Health Med.

[43] Alzahrani SG, Watt RG, Sheiham A (2014). Patterns of clustering of six health-compromising behaviours in Saudi adolescents. BMC Public Health.

[44] Quintiliani L, Allen J, Marino M (2010). Multiple health behavior clusters among female college students. Patient Educ Couns.

[45] Dodd LJ, Al-Nakeeb Y, Nevill A (2010). Lifestyle risk factors of students: A cluster analytical approach. Prev Med.

[46] Lv J, Liu Q, Ren Y (2011). Socio-demographic association of multiple modifiable lifestyle risk factors and their clustering in a representative urban population of adults: A cross-sectional study in Hangzhou, China. Int J Behav Nutr Phys Act.

[47] Galan I, Rodriguez-Artalejo F, Tobias A (2005). Clustering of behavior-related risk factors and its association with subjective health. Gac Sanit.

[48] Kritsotakis G, Psarrou M, Vassilaki M (2016). Gender differences in the prevalence and clustering of multiple health risk behaviours in young adults. J Adv Nurs.

[49] Haug S, Schaub MP, Gross CS (2013). Predictors of hazardous drinking, tobacco smoking and physical inactivity in vocational school students. BMC Public Health.

[50] Idowu A, Fatusi AO, Olajide FO (2016). Clustering of behavioural risk factors for non-communicable diseases (NCDs) among rural-based adolescents in south-west Nigeria. Int J Adolesc Med Health.

[51] Lippke S, Nigg CR, Maddock JE (2012). Health-promoting and health-risk behaviors: Theory-driven analyses of multiple health behavior change in three international samples. Int J Behav Med.

[52] Nigg CR, Lee H, Hubbard AE (2009). Gateway health behaviors in college students: Investigating transfer and compensation effects. J Am Coll Health.

[53] Costa FM, Jessor R, Turbin MS (2007). College student involvement in cigarette smoking: The role of psychosocial and behavioral protection and risk. Nicotine Tob Res.

[54] Raynor DA, Levine H (2005). Associations between the five-factor model of personality and health behaviors among college students. J Am Coll Health.

[55] Busch V, Van Stel HF, Schrijvers AJ (2013). Clustering of health-related behaviors, health outcomes and demographics in Dutch adolescents: A cross-sectional study. BMC Public Health.

[56] Li Y, Feng X, Zhang M (2016). Clustering of cardiovascular behavioral risk factors and blood pressure among people diagnosed with hypertension: A nationally representative survey in China. Sci Rep.

[57] Jawad M, Lee JT, Millett C (2015). Waterpipe tobacco smoking prevalence and correlates in 25 Eastern Mediterranean and Eastern European countries: Cross-sectional analysis of the Global Youth Tobacco Survey. Nicotine Tob Res.

